# Exuberant Vasospastic Angina Simulating Severe Three-Vessel Disease

**DOI:** 10.5935/abc.20170071

**Published:** 2017-06

**Authors:** Bruno Marmelo, Luís Abreu, Júlio Gil, Pedro Ferreira, José Cabral

**Affiliations:** Centro Hospitalar Tondela-Viseu

**Keywords:** Angina, Stable / complications, Coronary Vasoespasm, Acute Coronary Syndrome, Coronary Angiography

A 56-year-old Caucasian male, came to our hospital complaining of thoracic oppression at exertion and sometimes occurring at rest, lasting for a few minutes. The patient was an active smoker, with a moderate alcohol consumption habit and had had an episode of unstable angina two months earlier. At that episode, two drug-eluting stents were implanted, one in the distal anterior descending artery and the other in the proximal first diagonal artery. The ECG showed mild ST-elevation in V1-V3 and a T-wave inversion in V3-V5. There was a slight increase in Troponin I up to 0.24 ng/mL but the blood tests were otherwise unremarkable. The patient was admitted at the coronary unit and was scheduled for urgent coronary angiogram. The exam revealed severe and diffuse stenosis in the territories of the right and left coronary arteries with slow flow (TIMI 1-2), sparing only the stented segments (picture/video 1). The administration of 2 mg of intracoronary isosorbite dinitrate reverted all the stenosis but slow flow (TIMI 2) was still observed in the left coronary artery. Hence, the diagnosis of vasospastic angina was made. The patient was successfully controlled with calcium antagonists and has remained asymptomatic.

Vasospastic angina is commonly misinterpreted as acute coronary syndrome. Although its pathophysiology is not fully understood, it usually has a favorable long-term prognosis, although coronary artery spasms may have an important role in arrhythmia generation and subsequent cardiac arrest.

**Figure 1:**
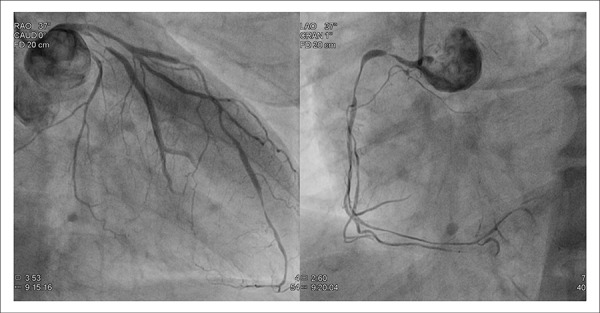
Left and right coronary angiogram showing multiple severe stenosis and slow flow.

**Video 1:** Left and right coronary angiogram showing multiple severe stenosis and slow flow followed by administration of intracoronary isosorbide dinitrate and stenosis resolution. Access the video through the link: http://www.arquivosonline.com.br/2017/english/10806/video_ing.asp

